# Functional analysis of *Leifsonia xyli* subsp*. xyli* membrane protein gene *Lxx18460* (anti-sigma K)

**DOI:** 10.1186/s12866-018-1378-2

**Published:** 2019-01-07

**Authors:** Kai Zhu, Min Shao, Dan Zhou, Yong-Xiu Xing, Li-Tao Yang, Yang-Rui Li

**Affiliations:** 1 College of Agriculture, State Key Laboratory of Conservation and Utilization of Subtropical Agro-bio resources, Guangxi University, Nanning, 530005 China; 2 Ministry of Agriculture Key Laboratory of Sugarcane Biotechnology and Genetic Improvement (Guangxi), Guangxi Key Laboratory of Sugarcane Genetic Improvement, Sugarcane Research Center, Chinese Academy of Agricultural Sciences-Guangxi Academy of Agricultural Sciences, Nanning, 530007 China

**Keywords:** Ratoon stunting disease, *Lxx18460*, Transgenic tobacco, Transcriptome, Functional analysis

## Abstract

**Background:**

Sugarcane is an important sugar and economic crop in the world. Ratoon stunting Disease (RSD) of sugarcane, caused by *Leifsonia xyli* subsp. *xyli*, is widespread in countries and regions where sugarcane is grown and also limited to sugarcane productivity. Although the whole genome sequencing of *Leifsonia xyli* subsp. *xyli* was completed, progress in understanding the molecular mechanism of the disease has been slow because it is difficult to grow in culture.

**Results:**

The *Leifsonia xyli* subsp. *xyli* membrane protein gene *Lxx18460* (anti-sigma K) was cloned from the *Lxx*-infected sugarcane cultivar GT11 at the mature stage using RT-PCR technique, and the gene structure and expression in infected sugarcane were analyzed. The *Lxx18460* gene was transformed into *Nicotiana tabacum* by *Agrobacterium tumefaciens*-mediation. The transgenic tobacco plants overexpressing *Lxx18460* had lower levels in plant height, leaf area, net photosynthetic rate and endogenous hormones of IAA, ABA and GA_3_, as well as lower activities of three antioxidant enzymes, superoxide dismutase (SOD), peroxidase (POD) and catalase (CAT) than the wild type (WT) tobacco. With the plant growth, the expression of *Lxx18460* gene and protein was increased. To better understand the regulation of *Lxx18460* expression, transcriptome analysis of leaves from transgenic and wild type tobacco was performed. A total of 60,222 all-unigenes were obtained through BGISEQ-500 sequencing. Compared the transgenic plants with the WT plants, 11,696 upregulated and 5949 downregulated genes were identified. These differentially expressed genes involved in many metabolic pathways including signal transduction, biosynthesis of other secondary metabolism, carbohydrate metabolism and so on. Though the data presented here are from a heterologous system, *Lxx 18460* has an adverse impact on the growth of tobacco; it reduces the photosynthesis of tobacco, destroys the activity of defense enzymes, and affects the levels of endogenous hormones, which indicate that *Lxx18460* may act important roles in the course of infection in sugarcane.

**Conclusions:**

This is the first study on analyzing the function of the membrane protein gene *Lxx18460* of anti-sigma K (σK) factor in *Leifsonia xyli* subsp. *xyli*. Our findings will improve the understanding of the interaction between the RSD pathogen *Leifsonia xyli* subsp. *xyli* and sugarcane. The output of this study will also be helpful to explore the pathogenesis of RSD.

**Electronic supplementary material:**

The online version of this article (10.1186/s12866-018-1378-2) contains supplementary material, which is available to authorized users.

## Background

Sugarcane is an important economic crop in Guangxi province which contributes more than 60% of the total sugar production in China [1, 2]. Ratoon stunting disease (RSD), caused by a nutritionally fastidious Gram-positive bacterium (*Leifsonia xyli* subsp. *xyli* – *Lxx*), is widespread in worldwide sugarcane production areas [3, 4] and responsible for substantial losses in yield [5–8]. RSD control measures include meristem tissue culture, sterilization of harvesting and planting equipment and seedcane heat treatment. However, all of these measures have limited efficacy, RSD persists in sugarcane as a major threat to sugar industry.

*Lxx* colonizes at xylem vessels, mesophyll and bundle sheath cells surrounding the vascular system [9, 10]. *Lxx* is genetically uniform, and it has no genetic variation [7, 11]. The whole genome sequencing of *Lxx* (CTCB07) was completed by Monteiro-Vitorello et al. [12]. The sequencing results have caused research interests in host recognition, pathogenicity gene and infection mechanism. Two possible pathogenicity genes, that is, pectinase and cellulose genes would be involved in cell wall degradation, but no evidence of this in the electron micrographs has been shown in the literature [13, 14]. Other pathogenicity genes may be involved in the production of abscisic acid, which inhibits growth of sugarcane [12]. No any definite disease-causing gene has been found up to now.

*Lxx* is a bacterium, σ factor is one of protein subunits (α_2_ββ’σ) of the bacterial RNA polymerase holoenzyme and also a major regulatory factor of prokaryotic gene expression regulation. It can be used to identify the target gene promoter regions, promote the combination of the target region and RNA polymerase holoenzyme [15], and anti σ factors could suppress the transcription by affecting the σ factor failing to identify the promoter protein conformation or failing to form RNA polymerase holoenzyme. The first anti σ factor AsiA was cloned in 2002 [16], and since then many anti σ factors have been cloned subsequently [17, 18]. A transmembrane anti σ factor RseA is the product of co-transcription by downstream genes σ^ECF^ and σ^E^ in *Escherichia coli*, which tightly binds to σ^E^, then restricts its identification of promoter and its binding with the core enzyme of RNA polymerase [19]. Rv0444c of *Mycobacterium tuberculosis* encodes anti-Sigma K factor (RskA), which could inhibit the Sigma K regulating action and affect the antigen protein expression of MPB70 and MPB83 ultimately [20]. The σR factor (sigma R factor, SigR) activity is regulated by the anti σ factor (regulator of sigma R, RsrA) of genus *Streptomyces* [21]. Previous studies showed that there are huge differences among anti σ factors in sequence and structure, and these differences led the anti σ factors to react to stimulus response and improve flexibility in bacterial transcriptional regulation [22].

Transcription is the first step of gene expression, and σ factor plays a key role in starting transcription, so studies on σ factor and anti σ factor are considered the key for regulating the gene expression in recent years. However, it is hard to culture *Lxx* in vitro, and the pathogenesis research on RSD is still at a preliminary stage. The anti σ factors of *Lxx* and their corresponding features and functionalities have not been sufficiently analyzed. In this study, we isolated and cloned the membrane protein gene *Lxx18640* (anti-sigma K factor, RskA) of *Lxx*, analyzed its biological characteristics, detected its expression in infected sugarcane, and verified its expression in a heterologous system, to further discern its functional role.

## Results

### Molecular characterization and bioinformatics analysis of *Lxx18460*

A full-length *Lxx18460* cDNA was isolated from the infected sugarcane. It was 720 bp in size and the predicted molecular weight of Lxx18460 protein was 24.8 kDa with an isoelectric point (pI) of 5.01 (see Additional file 1). The sequencing results showed that its homology with the *Lxx* (NC_006087) from GenBank was up to 100%, and has been registered with the accession No. JQ740153.

**Fig. 1:**
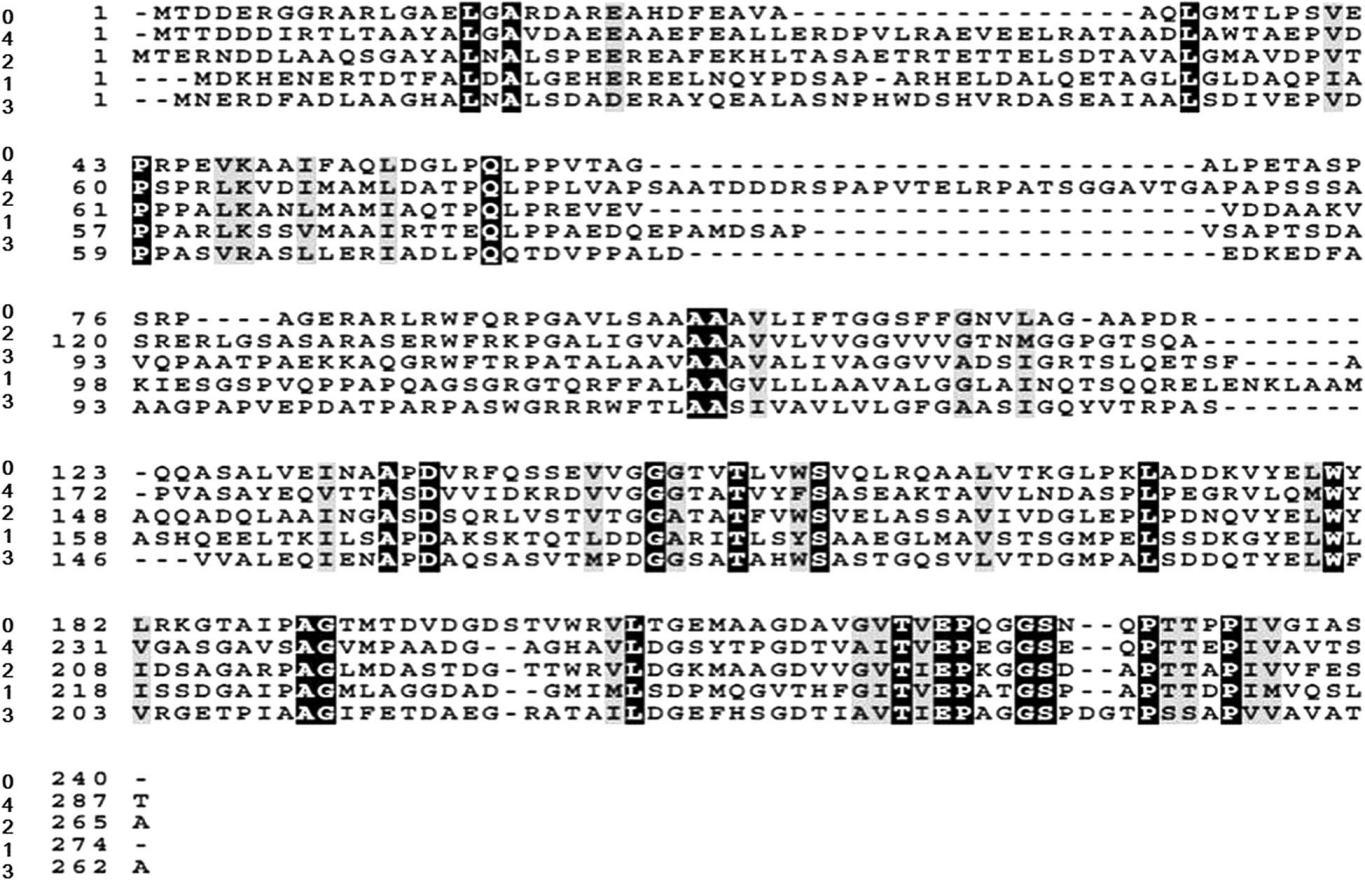
Multi-alignment of amino acid of the RskA proteins isolated from five bacterial strains. The alignment was performed using vector NTI Advance software. Black backgrounds represent identical amino acid residue sequences and dashed lines represent gaps introduced for optimal alignment. 0: *Lxx18460*; 1: *Arthrobacter arilaitensis* Re117 (WP_013348385.1); 2: Marine actinobacterium PHSC20C1 (WP_009772989.1); 3: *Microbacterium testaceum* StLB037 (WP_013585798.1); 4: *Clavibacter michiganensis* subsp. *michiganensis* NCPPB382 (WP_012039198.1)

There is a conserved domain in the deducted Lxx18460 protein through NCBI CDD (Conserved Domain Databases) (see Additional file 1). The results of functional analysis of *Lxx*18460 using software of SMART and Motif Scan showed that the Lxx18460 protein domain located at the 43–238 position is RskA family (anti ơ K factor). The prediction of the transmembrane region of Lxx18460 shows that there is a transmembrane region, which is located between 92 and 114 bp. The amino acid sequence homology of Lxx18460 is low as compared to other strains (Fig. 1). BLAST analysis indicated that Lxx18460 had 34% identity to *Arthrobacter arilaitensis*, 42% to marine actinobacterium PHSC20C1, 41% to *Microbacterium testaceum*, and 39% to *Clavibacter michiganensis* subsp. *michiganensis*, respectively. The results suggested that the anti ơ factor has huge differences in structure and sequence in different *Leifsonia* xylem strains.

### Expression of *Lxx18460* gene in *Lxx*-infected sugarcane

**Fig. 2:**
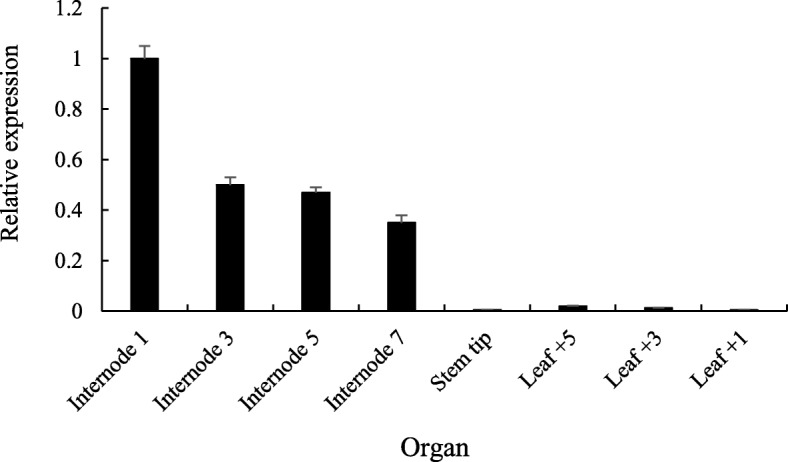
Expression of *Lxx18460* gene in stalks and leaves of sugarcane infected with *Lxx* at maturing stage

Real-time PCR results showed that the *Lxx18460* gene was expressed in *Lxx*-infected sugarcane stalks and leaves at mature stage, however, for different tissues, the relative expression level was different in different organs (Fig. 2). The highest relative expression was found in internode 1, and there was no obvious difference between internode 3 and internode 5. The relative expression level showed decreasing gradually from internode 1 to stem tip where it was almost zero. The relative expression level was lower in leaves than stems, and it decreased gradually from leaf + 5 to leaf + 1.

### Transgenic expression of *Lxx18460* in tobacco

**Fig. 3:**
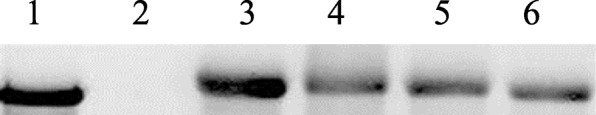
Western blot of transgenic tobacco lines transformed with *Lxx18460* monoclonal antibody. Lines from left to right: lane 1, positive control (purified Lxx18460 protein); lane 2, WT tobacco plant; lanes 3–6, transgenic plants

Forty-five independent putative transgenic tobacco calli were produced after co-cultivation of wild-type calli with *Agrobacterium tumefaciens* carrying *Lxx18460* in its T-DNA. The twenty-one putative transgenic plants regenerated were screened on a medium containing kanamycin (100 mg L^− 1^), and fifteen of them were found having *Lxx18460* gene, *NPTII* by PCR detection (see Additional file 2). The result of PCR analysis indicated that the co-transformation rate of *Lxx18460* and *NPTII* genes was approximately 71%. The transgenic and WT tobacco plants were further detected with Western Blot using Lxx18460 monoclonal antibody and the results showed that the *Lxx18460* gene had been successfully integrated into the transgenic plants and expressed (Fig. 3). Simultaneously, we got the correct sizes of *Lxx18460* and *NPTII* fragment in all the transgenic plants, and found that the *Lxx18460* and the *NPTII* gene existed in all the transgenic plants but not in WT tobacco.

**Fig. 4:**
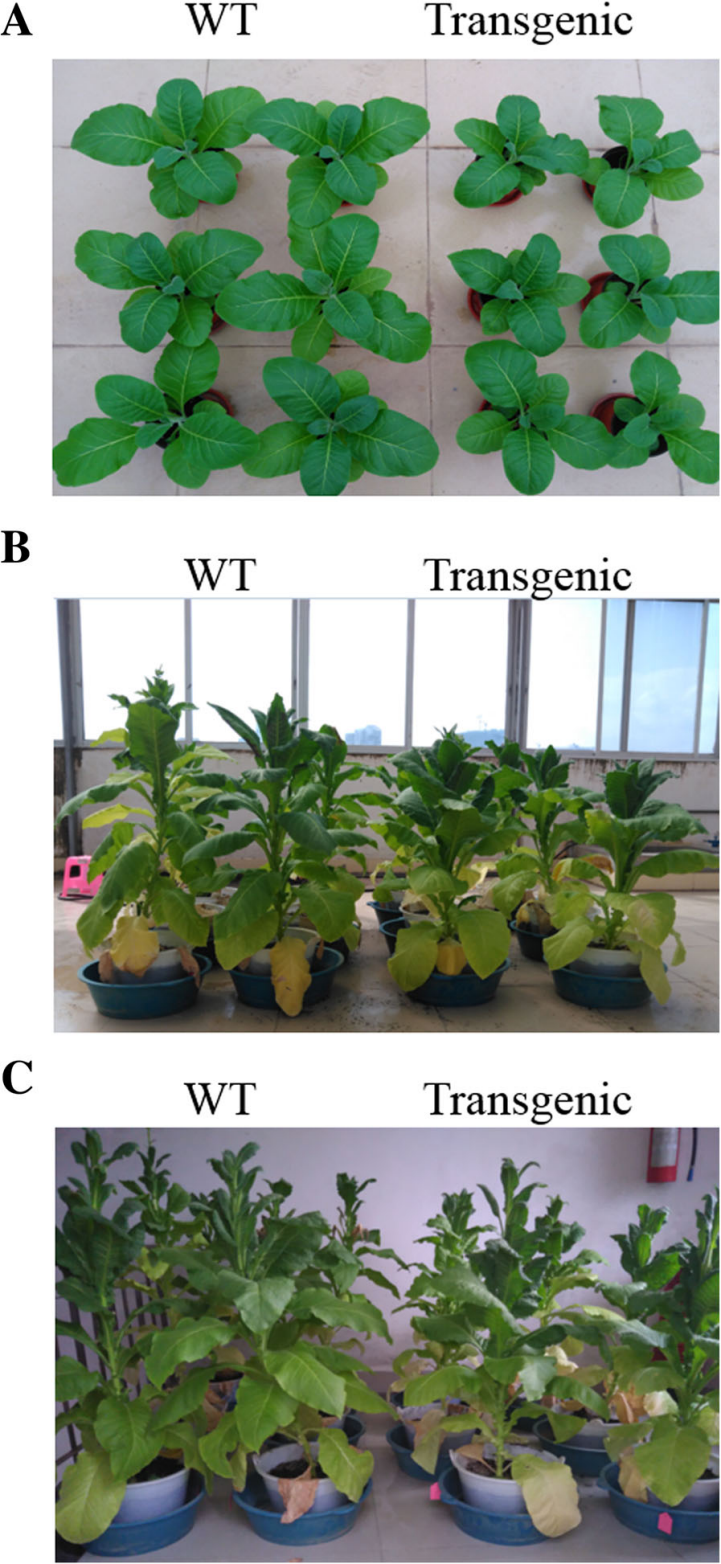
Phenotypes of *Lxx18460* transgenic and WT tobacco plants. **a**, **b** and **c** *Lxx18460* transgenic and WT plants at 50, 80 and 110 days after emergence, respectively

The WT and transgenic plants showed big difference in phenotype (Fig. 4). Compared with WT, the transgenic tobacco showed shorter plant height, finer stem, smaller leaf with slightly fold on the edge. These results suggest that the expression of *Lxx18460* might have affected the growth of the transgenic plants.

### Expression of *Lxx18460* gene decreased plant height, leaf area and net photosynthetic rate in tobacco

**Fig. 5:**
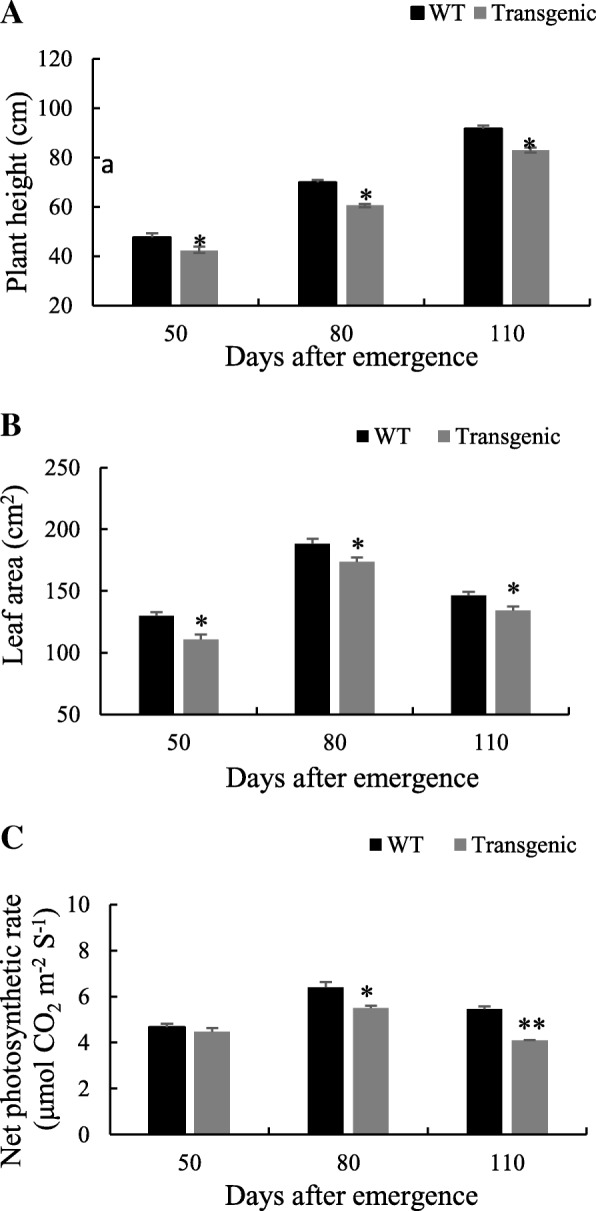
Plant height (**a**), leaf area (**b**), and net photosynthetic rate (**c**) in transgenic and WT tobacco plants. Tobacco leaf samples were taken at 50, 80 and 110 days after emergence. All the data are means ± SD calculated from six replicates. Symbols * and ** indicate significant difference between lines at 0.05 and 0.01 levels, respectively. Six biological experiments were performed, which produced similar results

The plant height, leaf area and net photosynthetic rate in WT and transgenic tobacco (TT) were compared. As shown in Fig. 5a and b, the plant height and leaf area were lower in TT than in WT from 50 to 110 days after emergence, and there were obvious differences between TT and WT. Net photosynthetic rate was higher in WT than that in TT (Fig. 5c).

### Expression of *Lxx18460* gene declined the SOD, POD, CAT and T-AOC activities in tobacco leaves

**Fig. 6:**
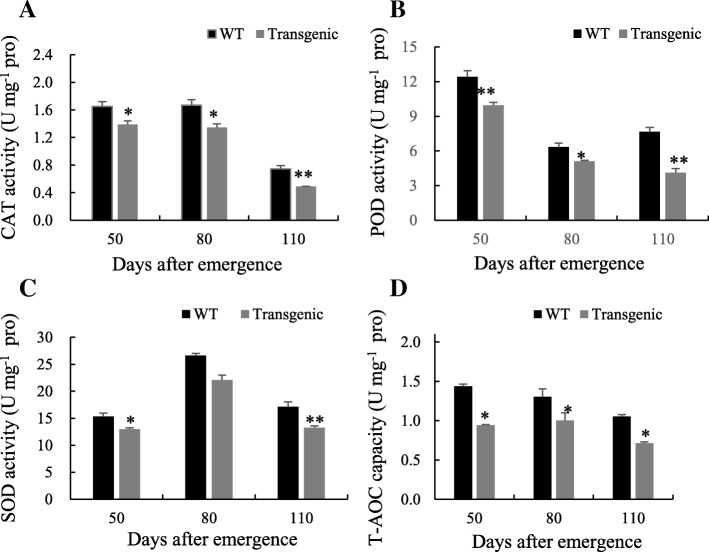
Activities of SOD, POD, CAT and T-AOC in transgenic and WT tobacco leaves. Tobacco leaf samples were taken at 50, 80 and110 days after emergence. Data are means ± SD calculated from six replicates. Symbols * and ** indicate significant difference between lines at 0.05 and 0.01 levels, respectively. **a**, CAT activity; **b**, POD activity; **c**, SOD activity; **d**, T-AOC capacity

The SOD, POD, CAT and T-AOC activities in tobacco leaves were measured at different time. The results showed that the CAT, POD and SOD activities were lower in TT than WT plants (Fig. 6a-c). As compared to TT, the T-AOC activity was significantly higher in WT, suggesting that the total antioxidant capacity is weaken in TT and stronger in WT (Fig. 6d).

### IAA, ABA and GA_3_ contents in tobacco leaves

**Fig. 7:**
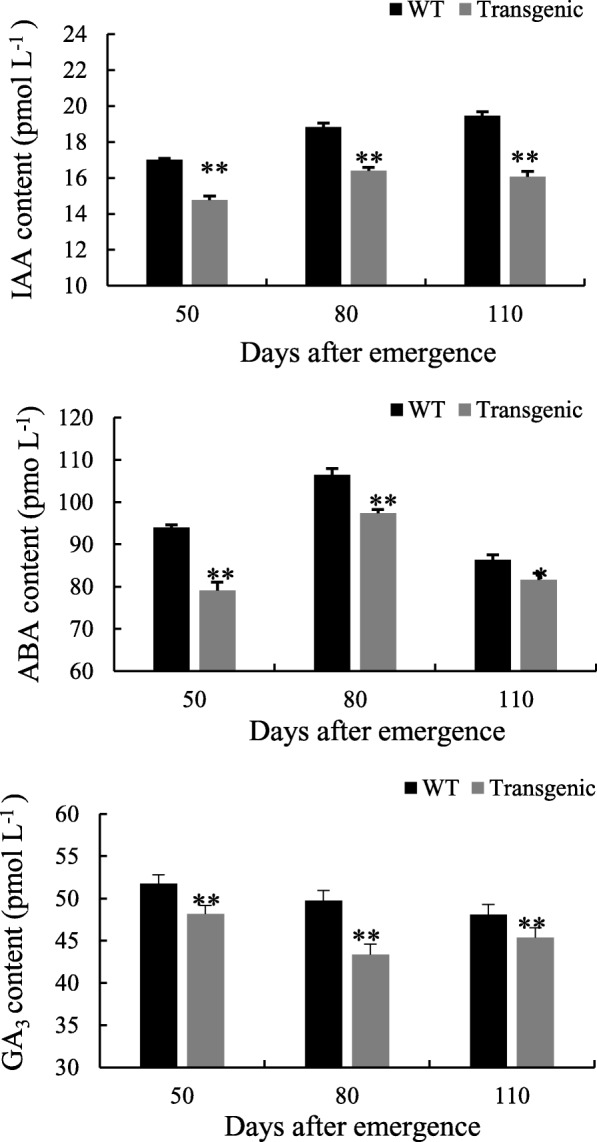
IAA, GA_3_ and ABA contents in leaves of transgenic and WT tobacco plants. The measurements were repeated for five times at each sampling date. Data are means ± SD calculated from three replicates. Symbols * and ** indicate significant difference between lines at 0.05 and 0.01 levels, respectively. Six separate experiments were performed, which produced similar results. **a** IAA content; **b** ABA content and; **c** GA_3_ content

Compared with the WT, the IAA, ABA and GA_3_ contents in leaves were significantly lower in the transgenic tobacco plants (Fig. 7).

### *Lxx18460* gene expression in transgenic tobacco plant

**Fig. 8:**
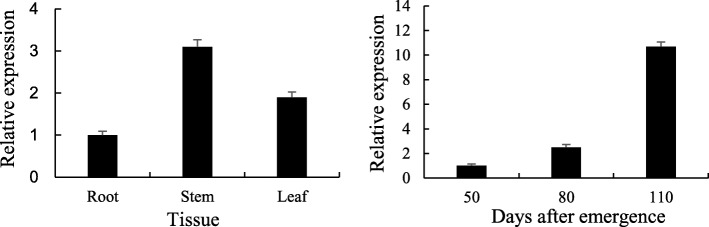
Relative expression of *Lxx18460* gene in transgenic tobacco plants. **a**, Relative expression of *Lxx18460* gene in root, stem and leaf of transgenic tobacco plants at 110 day after emergence. **b**, Relative expression of *Lxx18460* gene in transgenic tobacco leaves in different days after emergence

The *Lxx18460* gene expression in transgenic tobacco was detected with qRT-PCR, and the results showed that the relative expression of *Lxx18460* gene was increasing with plant age and significantly higher in stalk and at 110 day after emergence (Fig. 8).

### Lxx18460 protein expression in transgenic tobacco plant

**Fig. 9:**
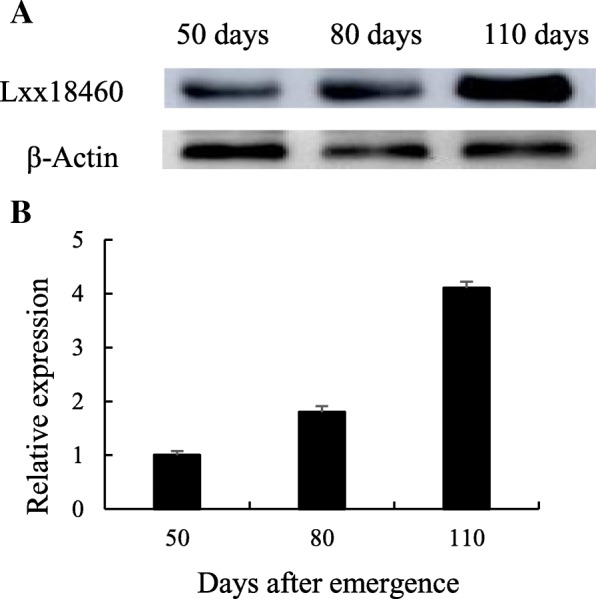
Lxx18460 protein expression in transgenic tobacco plant. **a**, Western blot of Lxx18460 protein in transgenic tobacco leaves and; **b**, Expression of Lxx18460 protein in transgenic tobacco leaves in different days after emergence

The Lxx18460 protein expression in the transgenic tobacco was detected with Western Blot, and the results (Fig. 9a) showed that the relative expression of Lxx18460 protein was accorded with the *Lxx18460* gene expression in the transgenic tobacco plant, which was increasing with plant age and significantly higher at 110 day after emergence (Fig. 9b).

### Transcriptome analysis

**Fig. 10:**
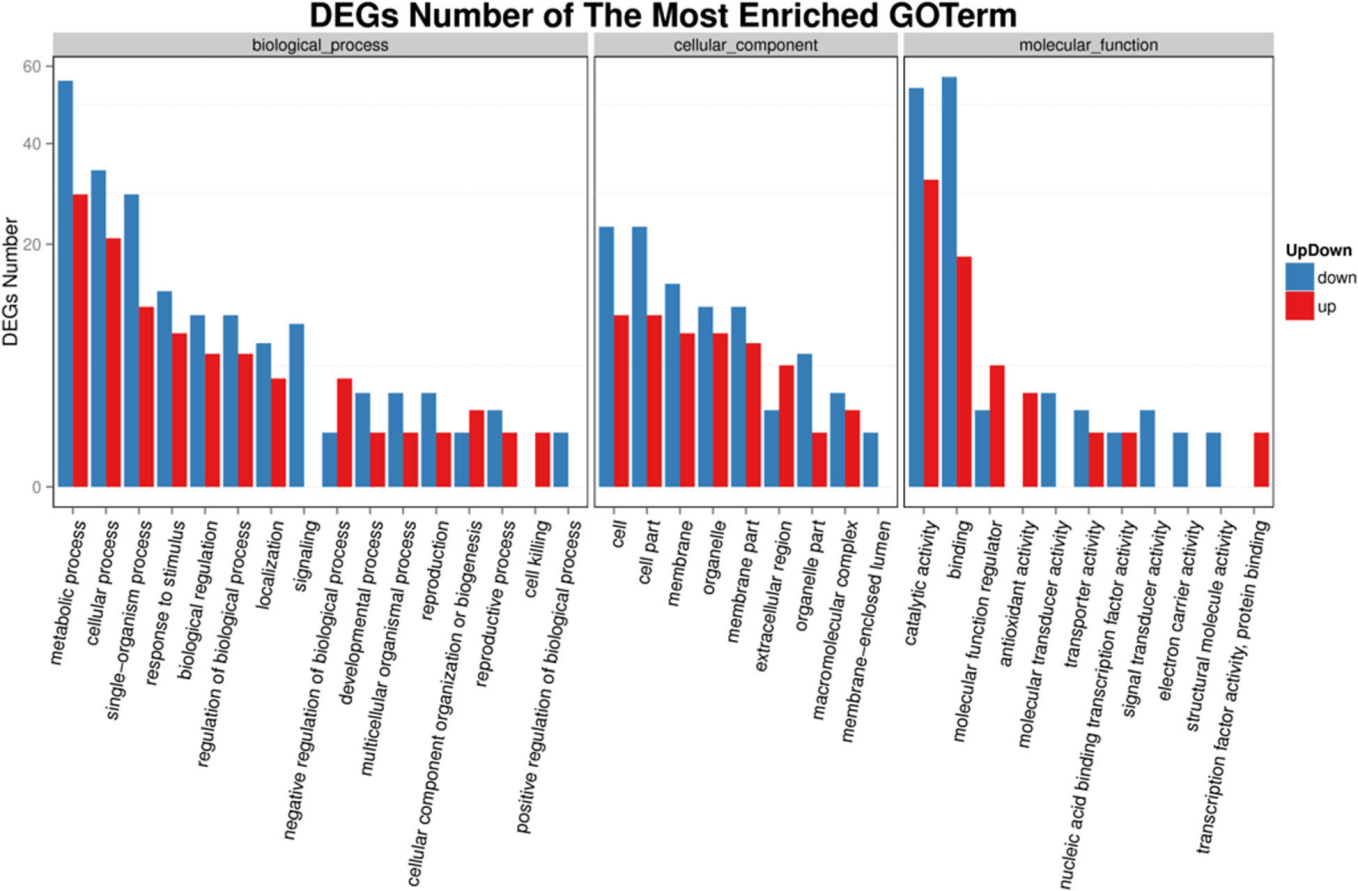
Differential gene GO functional classification map compared *Lxx18460* transgenic with WT tobacco plants

The transcriptome sequencing was performed using BGISEQ-500 platform, and total 60,222 genes were detected. The FPKM method was used to identify the genes with different expression levels. The results showed that a total of 17,645 genes were significantly changed. Among these genes, 11,696 genes were upregulated and 5949 genes downregulated after 110 days transforming *Lxx18460*. The differentially expressed genes were subjected into GO enrichment analysis to characterize the major biological functions. The most common terms were “metabolic process”, “cell”/“cell part”, and “catalytic activity” in each of the three main categories (biological process, cellular component, and molecular function), respectively. It was noticed that there were downregulated genes only in the terms “signal transduction”, “positive regulation of biological process”, “membrane enclosed lumen”, “molecular transducer activity”, “electron carrier activity”, and “structural molecule activity”. There were upregulated genes only in the terms “cell Killing”, “transcription factor activity”, and “protein binding” (Fig. 10). KEGG pathway analysis showed that these differentially expressed genes were involved in ABC transporters, RNA polymerase, fatty acid degradation, nitrogen metabolism, secondary metabolite synthesis and so on (see Additional file 3). Quantitative real-time PCR was performed on a subset of 8 randomly selected differentially expressed genes to validate the expression data from the BGISEQ RNA-seq analysis. These genes were involved in plant biometabolism, photosynthesis or plant defense reactions, and also included upregulated and downregulated unigenes (see Additional file 4). The expression patterns for all the 8 genes were in agreement with the RNA-seq data. The transcriptomics data have been deposited in NCBI with the accession No. PRJNA498357.

## Discussion

In this study, the full-length *Lxx18460* gene, the anti ơ factor of *Lxx* was cloned and characterized. It was found that the amino acid sequence has low homology with those from other strains, so it is speculated that most ơ factor homology is not high between different bacterial strains. Since the anti ơ factor has huge differences in structure and sequence, the bacteria could respond to the stimulus elements, which could better regulate certain gene transcriptions and expressions. Studies have shown that the concentration of *Lxx* in sugarcane is different in various tissues, and the *Lxx* titers decreasing from the basal part to the tail of stalk in sugarcane [23]. Therefore, the expression of *Lxx18460* in infected plant is related to the level of *Lxx* in different organs of sugarcane. We used the purified protein of Lxx18460 to obtain monoclonal antibody, which can be used for qualitative and quantitative detection of Lxx18460 protein. In addition, it can be used to further explore the colonization of the bacterium-*Lxx* at transcription and translation levels using *Lxx18460* gene.

Li et al. [24] constructed the eukaryotic expression vector of *TA3–13*, which is a gene coding for the fragment of a cold shock protein WCP1 of wheat (*Triticum aestivum* L.), and transformed it into tobacco; the transgenic tobacco showed significant resistance against *Tobacco mosaic virus* and rot pathogen *Pectobactrium carotovorum* subsp. *carotovorum*. Tang et al. [25] transformed *Sugarcane mosaic virus* (ScMV) coat protein (*CP*) gene into sugarcane variety Badila, and the transgenic sugarcane of *ScMV-CP* gene significantly enhances the *ScMV* resistance of sugarcane and improve the cane yield and quality greatly. In this study, we analyzed the function of *Lxx* membrane protein gene *Lxx18460* (anti-sigma K) by transforming it into tobacco, and then investigated the phenotypic, physiological, biochemical and molecular changes in the transgenic tobacco plants. We found that the net photosynthetic rate was lower in the transgenic tobacco than that in WT plants. The reason may be that *Lxx18460* gene inhibited the expression of photosynthetic enzyme genes, and this speculation was confirmed by quantifying the relative expression of photosynthesis related genes using RT-PCR. We observed that the expression of *Rubisco* and *PEPC* genes were inhibited in the transgenic tobacco. Yu et al. [26] reported that the net photosynthetic rate in tomato decreased after infected with tomato yellow leaf curl virus. Sun [27] found that 51 down-regulated genes coded the proteins involved in photosynthesis in rice after blast infection, and the pathogen suppressed the photosynthesis genes’ expression and decreased the photosynthesis greatly. Plant could form an antioxidant protection mechanism to remove excess free radicals, and the defense enzymes of SOD, POD and CAT play a very important role in scavenging reactive oxygen species (ROS) [28–30]. Excessive expression of SOD enzyme in transgenic tobacco can increase the tolerance under stress [31]. POD activity in alfalfa increased after infection by *brownblotch* strains [32], while CAT activity in tobacco increased after infected by *pseudomonas solanacearum* [33]. In this study, the SOD, POD and CAT and T-AOC activities in the *Lxx18460* transgenic tobacco declined as compared to the WT plants, which suggested that the defense capability had been decreased by transformation of *Lxx18460* gene. The transcriptome analysis results also showed that the expression of catalase was decreased. The transgenic plants were more susceptible to membrane damage compared to the WT plants. The pathogen *Lxx* showed extremely strong infection effect though it had been diluted for hundreds of times [34]. It might be possible that at the beginning of the infection, the expression of *Lxx18460* gene in pathogenic bacteria inhibits the gene transcription of the defense enzymes and damage the defense regulation of host plants, which would help the pathogen to infect the host and to multiply inside the plant cell. Growth of plant is not only affected by the change of various enzyme activities, but also regulated by various endogenous hormones [35]. According to the results of this study, the syntheses of GA_3_, IAA and ABA were inhibited in the *Lxx18460* transgenic plants, which suggested that the expression of *Lxx18460* gene suppresses the biosynthesis and signaling pathways of the endogenous hormones. The physiological and phenotypic changes in the transgenic tobacco proved that *Lxx18460* plays a role in inhibiting the signal transduction and hormone synthesis gene expression.

It has been reported that the photosynthesis and cane yield were decreased, and defense enzyme activities and endogenous hormone levels were adversely affected by the infection of *Lxx* [36–39]. Interestingly, the symptoms of *Lxx18460* gene expression in tobacco are similar to those caused by *Lxx* in sugarcane. After transforming the gene *Lxx18460* into tobacco, the net photosynthetic rate was decreased and the endogenous hormone synthesis inhibited, and the growth of transgenic plants hindered. As the result the plant height was lower and the leaf area smaller, subsequently, the plants become dwarf and smaller, and the biomass decreased. Furthermore, this study demonstrated that the expression level of *Lxx18460* gene and Lxx18460 protein was stably increased with time course during plant growth. The results strongly supported the view that the *Lxx18460* gene had been transformed into tobacco, and its expression had slowed down the growth of the transgenic tobacco plants. The higher expression showed the *Lxx18460* gene, the more obvious was the effect on transgenic tobacco.

## Conclusion

The membrane protein gene *Lxx18460* of anti-sigma K (ơK) factor in *Leifsonia xyli* subsp. *xyli* in sugarcane was cloned, and its structure and expression in sugarcane infected by RSD were analyzed. The function of *Lxx18460* gene was analyzed by transforming it into tobacco. The results showed that the expression of *Lxx18460* gene could inhibit the growth of the transgenic tobacco. *Lxx18460* gene plays an important role in regulating signal transduction, gene transcription and plant growth, and in the course of infection in sugarcane. It is important to further investigate the function of *Lxx18460* gene and its role in *Lxx* pathogen. This is the first report of analyzing the function of the membrane protein gene *Lxx18460* of anti-sigma K (σK) factor in *Leifsonia xyli* subsp. *xyli*. It is necessary to further investigate the interaction between sugarcane and *Lxx* using *Lxx18460* gene to illustrate the molecular mechanism of *Lxx* infection in sugarcane.

## Materials and methods

### Materials

The *Lxx*-infected sugarcane (*Saccharum* spp.) variety GT11 grown in the Sugarcane Institute, Guangxi University (Nanning, China, 108°19′E 22°49’N) was used for cloning *Lxx18460*. The wild type tobacco (*Nicotiana tabacum* L. variety 346) was used for transgenic analysis.

### Cloning and sequencing of *Lxx18460* gene

Total RNA from the *Lxx*-infected sugarcane stalk was extracted using Trizol Reagent (Cowin Biosciences, Beijing, China) [40], and the cDNA template for RACE-PCR amplification was prepared according to the User Manual (TaKaRa, Dalian, China). Based on the sequence of *Lxx18460* from the GenBank (NC-006087), we design the specific primers as R1: 5’-ATGACCGACGACGAGCGGGG-3′ and R2: 5’ TCAGGAGGCGATGCCTACGA-3′ using Primer Premier 5.0 software. The putative full length *Lxx18460* gene was obtained using RT-PCR technique. The reaction procedure was as follows: 10 min at 95 °C for degeneration, 40 cycles for 40 s at 94 °C, for 40 s at 62 °C, for 90 s at 72 °C, and a final extension for 10 min at 72 °C. The amplified target bands were recovered and purified with a Gel Extraction Kit (TaKaRa, Dalian, China), and then attached to pMD18-T vector (TaKaRa, Dalian, China), transformed into DH5a competent cells (Transgene, Beijing, China); and the positive clones were identified by sequencing.

BLAST (https://blast.ncbi.nlm.nih.gov/Blast.cgi) was used to analyze the sequence in the NCBI database. The physical properties of the *Lxx18460* protein were predicted using Expasy (http://web.expasy.org/protparam/). Conservative structure and function of the gene encoded protein domains were analyzed using NCBI CDD (Conserved Domain Databases), SMART (http://smart.embl-heidelberg.de/). Transmembrane structures of the protein were analyzed using TMPRED Server (http://embnet.vital-it.ch/software/TMPRED_form.html) and TMHMM2.0 (http://www.cbs.dtu.dk/services/TMHMM/).

### Quantitative real-time PCR (qRT-PCR) analysis of *Lxx18460* expression in *Lxx*-infected sugarcane and transgenic tobacco

Quantitative real-time PCR technique was applied to detect the expression of *Lxx18460* in different stalks and leaves during the mature growth stages. Internodes 1–7 represent the first to the seventh internode form the base of sugarcane, and leaves + 1 to leaf + 5 are the first to the fifth leaf with visible dewlap from sugarcane morphological upper part on the ground. The qRT-PCR was done according to the method outlined by Zhu et al. [41]. We calculated the relative level of gene expression using the 2^−ΔΔCt^ formula [42]. For transgenic tobacco, the *Lxx18460* gene expression was measured at 50 to 110 days after emergence. The *GAPDH* sugarcane gene (accession number EF189713) and *GAPDH* tobacco gene (accession number M14419) were used as the internal controls to quantify the relative transcript levels in *Lxx*-infected sugarcane and transgenic tobacco, respectively [43, 44].

### Lxx18460 protein prokaryotic expression, tobacco *Lxx18460* transformation and transgenic plant generation

The *Lxx18460* expression vector primers were designed according to the *Lxx18460* gene ORF. EcoR I endonuclease site, Xho I endonuclease site and protective base pair were added to the 5′ terminal of the primer for gene expression detection. The prokaryotic expression vector pET-30a was transformed into *E. coli* BL21(DE3)pLysS. After the IPTG induction process, the protein was isolated, purified and analyzed by SDS-PAGE with Image Lab 5.0 software. The protein was identified and confirmed by mass spectrometry (MALDI-TOF). The protein induced and over-expressed in *E. coli* was used as antigen to produce monoclonal antibody. ELISA test was applied to analyze the titer. The specificity was analyzed using Western blot in bacteria and in the healthy and *Lxx*-infected sugarcane plant samples.

The full length open reading frame (ORF) of *Lxx18460* was cloned using PCR technique, and then inserted into a pBI121 vector (conserved by our lab). This vector was under control of cauliflower mosaic virus 35S (CaMV35S) promoter and nopaline synthase (NOS) terminator [45]. The recombinant plasmid pBI121-*Lxx18460* construction was introduced into the *Agrobacterium tumefaciens* strain EHA 105, and then transformed into the WT tobacco callus as described by Horsch et al. [46].

The tobacco seeds were sterilized with 70% ethanol for 30 s and then rinsed with sterile ddH_2_O two times, followed by disinfection for 8–10 min in 4% sodium hypochlorite, rinsing with sterile ddH_2_O five times before they were sown in plate culture medium (M1) based on MS medium [47] supplemented with 25 g L^− 1^ sucrose and 0.7% agar (pH 5.8). The infected explants were co-cultured on M1 medium complemented with 1 mg L^− 1^ 6-benzyladenin (BA) and 0.5 mg L^− 1^ indoleacetic acid (IAA), which is defined as M2 medium, at 25 °C in dark for 4 days. Then the co-cultured plants were rinsed with ddH_2_O containing 300 mg L^− 1^ cephalosporin. Positive transgenic plants were selected on M2 medium containing 100 mg L^− 1^ of kanamycin. The kanamycin-resistant shoots were transferred to M3 selection medium (M1 medium plus 0.5 mg L^− 1^ IAA, 75 mg L^− 1^ kanamycin) for rooting. A few days later, the non-transgenic and transgenic status of the T1 plantlets was confirmed by PCR and Western blot analysis.

The T1 plants were just used for phenotypic observation. Seeds of wild type and the transgenic T1 plants were harvested and used for further experiments. The expression of *Lxx18460* in the independent T2 generation was also determined by PCR and Western blot. For physiological and biochemical analysis, the seedlings were grown in the glasshouse condition. During the T2 generation growth period, *Lxx18460* expression was observed and analyzed in the transgenic and WT plants using the same method as described above.

### PCR identification of transgenic tobacco

The genomic DNA was extracted from the leaves of transgenic and WT tobacco plants using a modified SDS extraction method [48], and tested for the presence of target gene of *Lxx18460* and marker gene of *NPTII*. The PCR reaction and PCR procedure was described in Additional file 5.

### Western blot analysis of Lxx18460 in transgenic tobacco

Total protein was extracted from the leaves of transgenic and WT tobacco plants using a modified phenol extraction method [49], and quantified by spectrophotometer. Western blot was processed as described by Zhu et al. [41].

### Plant height, leaf area and net photosynthetic rate measurements

Plant height was measured from the transgenic and WT tobacco stalk at 50, 80, and 110 days after emergence, respectively. Leaf area was measured using CI-203 Handheld Leaf Area Meter (CID Bio-Science, Inc., Camas, USA). Net photosynthetic rate was measured using Li-6400 photosynthesis system (LI-COR, Lincoln, USA).

### Activities of superoxide dismutase (SOD), peroxidase (POD), catalase (CAT) and total antioxidant capacity (T-AOC) assay

Activities of SOD, POD, CAT and T-AOC were measured from leaves of the transgenic and WT tobacco at 50, 80 and 110 days after emergence, respectively. The enzymes were extracted from 0.5 g leaf and grounded in 2 mL extraction buffer containing 0.1 M phosphate buffer (pH 7.4) and 1% polyvinylpyrolidone using a mortar and pestle kept in ice bath. The homogenate was centrifuged at 10,000×g for 10 min at 4 °C and the supernatant was used for enzyme assay. The SOD activity was measured using the nitro-blue tetrazolium method [50]. The activity of POD was measured using the guaiacol method [51]. The activities of CAT and T-AOC were measured using CAT and T-AOC Assay Kit (Jiancheng, Nanjing, China) according to the User Manual.

### Contents of IAA, ABA and GA_3_ assay

The fresh leaves of transgenic and WT tobacco were sampled at 50, 80 and 110 days after emergence, respectively. The plant hormone was extracted from 1 g leaf with 2 mL extract buffer (80% methanol containing 1 mM butylated hydroxytoluene) and grounded into homogenate in ice bath. Then solution containing 0.1% Tween-20 and 0.1% gelatin phosphate buffer, pH 7.5) was used to dissolve the hormone extract. The extracts were analyzed using ELISA method with five repeats [52].

### BGISEQ-500 sequencing and transcriptome analysis

Total RNA of each sample was extracted using the Trizol Reagent (Cowin Biosciences, Beijing, China) according to the manufacturer’s protocol from three biological replicates of each treatment (WT and Transgenic) resulting in 6 samples. The total RNA concentration of each sample was estimated using a Agilent 2100 Bioanalyzer (Agilent Technologies Inc.). The integrity of the RNA samples was assessed with 1.2% agarose gel electrophoresis. The six samples were sequenced using BGISEQ-500 Sequencing performed by Beijing Genomics Institute (BGI)-ShenZhen, China according to the manufacturer’s instructions. Detailed BGISEQ-500 sequencing experimental process and transcriptome data analysis method refer to Additional file 6.

### Data analysis

All the data were analyzed using Microsoft Excel 2003 (Microsoft) and IBM SPSS 21.0. All data points represent means from 15 plants of transgenic or WT tobacco. There were three technical replicates for each experiment, and statistical differences were compared based on the Duncan’s test with *P* values set at 0.05 and 0.01 levels, respectively, and means were compared by standard error (SE).

## Additional files

Additional file 1: Nucleotide, predicted amino acid sequences (A) and conserved domain (B) of Lxx18460. (DOCX 102 kb)

Additional file 2: Identification of transgenic tobacco plants detected with PCR. (DOCX 198 kb)

Additional file 3: KEGG pathway analysis of DEGs in *Lxx18460* transgenic tobacco. (DOCX 541 kb)

Additional file 4: Quantitative real-time PCR analysis of selected DEGs in *Lxx18460* transgenic tobacco. (DOCX 38 kb)

Additional file 5: PCR identification of transgenic tobacco. (DOCX 16 kb)

Additional file 6: BGISEQ-500 sequencing and transcriptome analysis, the primers of DEGs validated by qRTPCR analysis. (DOCX 21 kb)

## Abbreviations

ABA: Abscisic acid
BA: 6-benzyladenin
bp: Base pair
CAT: Catalase
cDNA: Complementary DNA
FPKM: Fragments per kilobase of transcript per million mapped reads
FW: Fresh weight
GA_3_: Gibberellic acid
GAPDH: Glyceraldehyde-3-phosphate dehydrogenase
IAA: Indoleacetic acid
*Lxx*: Leifsonia xyli subsp. Xyli
NCBI: National Center for Biotechnology Information
Nt: *Nicitiaba tabacum*
ơK: Anti-sigma K
ORF: Open reading frame
PEPC: Phosphoenolpyruvate carboxylase
pI: Isoelectric point
POD: Peroxidase
RACE: Rapid amplification of cDNA ends
ROS: Reactive oxygen species
RSD: Ratoon stunt disease
RT-PCR: Reverse transcription-polymerase chain reaction
SDS: Sodium dodecyl sulfate
SOD: Superoxide dismutase
TT: Transgenic tobacco
WT: Wild type
