# Supplementary figures and images for: Functional analysis of Leifsonia xyli subsp. xyli membrane protein gene Lxx18460 (anti-sigma K)
Source: BMC Microbiol. 2019 Jan 7;19:2. doi: 10.1186/s12866-018-1378-2 (PMC6323826; doi:10.1186/s12866-018-1378-2)

**A**


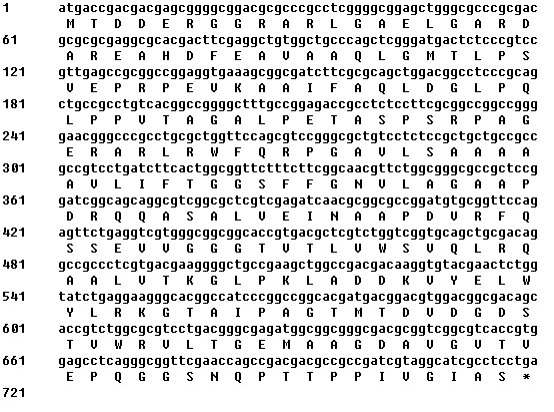


**B**


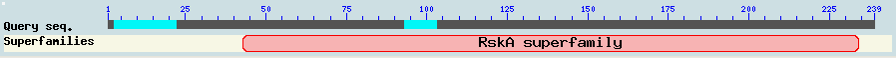


**Additional file 1** Nucleotide, predicted amino acid sequences (**A**) and conserved domain (**B**) of Lxx18460

Supplement: Supplementary file 1 — Nucleotide, predicted amino acid sequences (A) and conserved domain (B) of Lxx18460. (DOCX 102 kb) [file 12866_2018_1378_MOESM1_ESM.docx]
