# Supplementary material for: Functional analysis of Leifsonia xyli subsp. xyli membrane protein gene Lxx18460 (anti-sigma K)
Source: BMC Microbiol. 2019 Jan 7;19:2. doi: 10.1186/s12866-018-1378-2 (PMC6323826; doi:10.1186/s12866-018-1378-2)
Supplement: Supplementary file 2 — Identification of transgenic tobacco plants detected with PCR. (DOCX 198 kb) [file 12866_2018_1378_MOESM2_ESM.docx]

**A**

bp

M 1 2 3 4 5 6 7 8 9 10 11 12


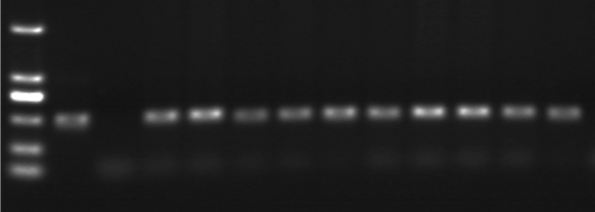


1000

385 bp

2000

750

**B**

M 1 2 3 4 5 6 7 8 9 10 11 12


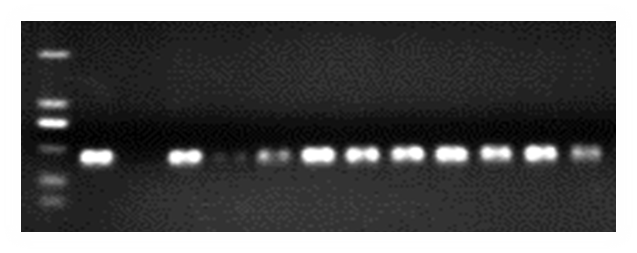


1000

500

2000

bp

327 bp

**Additional file 2** Identification of transgenic tobacco plants detected with PCR. **A**, Identification of *Lxx18460* gene. Lines from left to right: M, DL 2000 marker; lane 1, plasmid as positive control; lane 2, WT tobacco plant; lanes 3*–*12, transgenic plants. **B**, Identification of *NPTII* gene. Lines from left to right: M, DL 2000 marker; lane 1, plasmid as positive control; lane 2, WT tobacco plant; lanes 3–12, transgenic plants.
