# Supplementary material for: Functional analysis of Leifsonia xyli subsp. xyli membrane protein gene Lxx18460 (anti-sigma K)
Source: BMC Microbiol. 2019 Jan 7;19:2. doi: 10.1186/s12866-018-1378-2 (PMC6323826; doi:10.1186/s12866-018-1378-2)
Supplement: Supplementary file 3 — KEGG pathway analysis of DEGs in Lxx18460 transgenic tobacco. (DOCX 541 kb) [file 12866_2018_1378_MOESM3_ESM.docx]

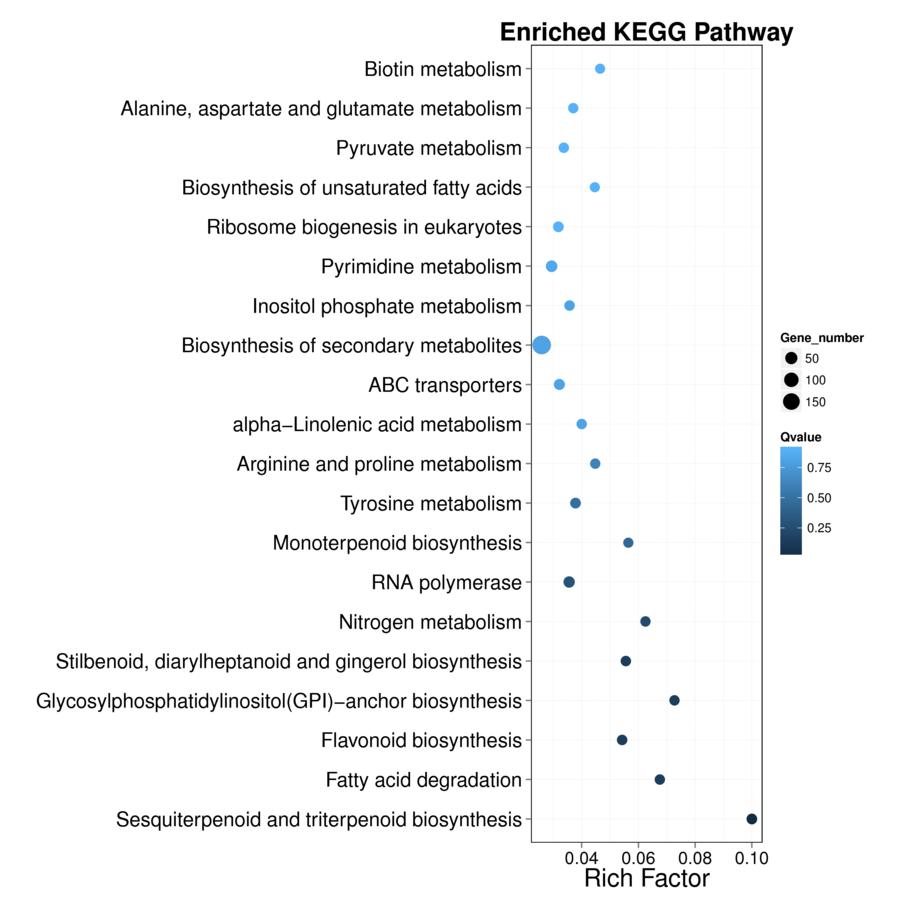


**Additional file 3** KEGG pathway analysis of differentially expressed genes in *Lxx18460* transgenic tobacco. The differentially expressed genes (log2 fold-changes) were significantly enriched in degradation of aromatic compounds and so on.
