# Supplementary material for: Functional analysis of Leifsonia xyli subsp. xyli membrane protein gene Lxx18460 (anti-sigma K)
Source: BMC Microbiol. 2019 Jan 7;19:2. doi: 10.1186/s12866-018-1378-2 (PMC6323826; doi:10.1186/s12866-018-1378-2)
Supplement: Supplementary file 4 — Quantitative real-time PCR analysis of selected DEGs in Lxx18460 transgenic tobacco. (DOCX 38 kb) [file 12866_2018_1378_MOESM4_ESM.docx]

**Additional file 4** Quantitative real-time PCR analysis of selected differentially expressed genes in *Lxx18460* transgenic tobacco.
