# Supplementary material for: Functional analysis of Leifsonia xyli subsp. xyli membrane protein gene Lxx18460 (anti-sigma K)
Source: BMC Microbiol. 2019 Jan 7;19:2. doi: 10.1186/s12866-018-1378-2 (PMC6323826; doi:10.1186/s12866-018-1378-2)
Supplement: Supplementary file 5 — PCR identification of transgenic tobacco. (DOCX 16 kb) [file 12866_2018_1378_MOESM5_ESM.docx]

**Additional file 5**

**PCR identification of transgenic tobacco**

The genomic DNA was extracted from the leaves of transgenic and WT tobacco plants using a modified SDS extraction method (Shan et al. 2011), and tested for the presence of the target gene *Lxx18460* and marker gene *NPTII*. The PCR reaction was performed in total 25 μL solution, containing 1 μL template DNA, 2 μL primer (10 μM), 9.5 μL RNase-free water and 12.5 μL Es Taq MasterMix (CWBIO, Beijing, China). The PCR system used for the *NPTII* gene was consisted of the initial denaturation at 94^o^C for 10 min, followed by 35 cycles of 94^o^C for 30 s, 68^o^C for 30 s, 72^o^C for 90 s, and a final 7 min elongation step at 72^o^C. For the target gene *Lxx18460*, the PCR procedure was consisted of initial denaturation at 95^o^C for 10 min, followed by 40 cycles of 94^o^C for 40 s, 62^o^C for 40 s, 62^o^C for 90 s, and a final 10 min elongation at 72^o^C. The PCR products were separated by electrophoresis on 1.0 % agarose gel and then observed under UV transmitted illumination.
