# Supplementary material for: Functional analysis of Leifsonia xyli subsp. xyli membrane protein gene Lxx18460 (anti-sigma K)
Source: BMC Microbiol. 2019 Jan 7;19:2. doi: 10.1186/s12866-018-1378-2 (PMC6323826; doi:10.1186/s12866-018-1378-2)
Supplement: Supplementary file 6 — BGISEQ-500 sequencing and transcriptome analysis, the primers of DEGs validated by qRTPCR analysis. (DOCX 21 kb) [file 12866_2018_1378_MOESM6_ESM.docx]

**Additional file 6**

**BGISEQ-500 sequencing and transcriptome analysis**

The first step in the workflow involved purifying the poly-A containing mRNA molecules using poly-T oligo-attached magnetic beads. The purified mRNA was fragmented into small pieces using divalent cations under elevated temperature. The cleaved RNA fragments were copied into the first strand cDNA using reverse transcriptase and random primers, followed by the second strand cDNA synthesis using DNA Polymerase I and RNase H. These cDNA fragments were added with a single 'A' base and subsequently ligated to the adapter. Then the products were purified and enriched with PCR amplification.

The PCR yield was quantified by Qubit and the samples were pooled together to make a single strand DNA circle (ssDNA circle), which gave the final library.

DNA nanoballs (DNBs) were generated with the ssDNA circle by rolling circle replication (RCR) to enlarge the fluorescent signals at the sequencing process. The DNBs were loaded into the patterned nanoarrays and pair-end reads of 100 bp were read through on the BGISEQ-500 platform for the following data analysis study. For this step, the BGISEQ-500 platform combines the DNA nanoball-based nanoarrays and stepwise sequencing using Combinational Probe-Anchor Synthesis Sequencing Method.

**Functional Annotation**

The unigene sequences were annotated by BLAST x alignment (e value < 0.00001) to protein databases such as the NCBI NR protein database (http://www.ncbi.nlm.nih.gov), the Swiss-Prot protein database (http://www.expasy.ch/sprot), the KEGG pathway database (http://www.genome.jp/kegg) and the COG database (http://www.ncbi.nlm.nih.gov/COG).

For the NR annotations, the Blast2GO program was used to obtain GO functional annotation, and the GO functional classification for all unigenes were obtained to understand the distribution of gene functions at the macroscopic level using the WEGO software. For COG annotations, the unigene sequences were aligned to the COG database to predict and classify possible functions. For pathway-enrichment analysis, all unigenes were mapped to terms in the KEGG pathway database.

**Differential Expression Analysis**

FPKM (Fragments per kilobase of transcript per million mapped reads) (Mortazavi et al. 2008) was used to calculate unigene expression levels. The FDR (False Discovery Rate) control method was applied to determine the threshold of p value in multiple tests and analyses (Reiner et al. 2003). After the FDR was obtained, the ratio of FPKM was used to calculate the fold-change in the expression of unigenes in six samples simultaneously. An FDR < 0.001 and the absolute value of log_2_Ratio ≥1 were used as the threshold for the judgment of the significance of the gene expression differences. The different expressed genes were then subjected to GO and KEGG Ontology enrichment analysis.

**Quantitative Real-Time PCR Analysis**

The selected unigenes were validated by quantitative real-time PCR. The first-strand cDNA was generated from the total RNA isolated from leaves of both wild type and the transgenic plants by using the Prime ScriptTM RT reagent Kit with gDNA Eraser (Takara, Dalian, China). The primers for qRT-PCR were designed using Primer 5.0 software. The glyceraldehyde-3-phosphate dehydrogenase (*GAPDH*) gene of tobacco was used as internal control. PCR amplifications were performed using a Roche Light Cycler 480 (Indianapolis, IN, USA) in a 20 μL reaction volume. Three independent biological replicates of each sample and three technical replicates of each biological replicate were used for qRT-PCR analysis. Relative gene expression levels were calculated using the 2^−∆∆Ct^ (Livak and Schmittgen 2001). The sequences of the primer sets used are listed as follows.

| Gene ID | Forward primer | Reverse primer | Gene description |
| --- | --- | --- | --- |
| 107772756 | TGCATCATCAACATGGAGGAC | ATGGATGGAGTTGGACCAGAA | Alcohol dehydrogenase |
| 107782278 | GTGTGAAAGCGCCTCCATGT | AAGAAAGAGGCTGGACCCCA | Zinc finger protein |
| 107821630 | GCTTCAGAACAGATGATGTAGTGC | TCACATGACCTCTAACTTCTCCAAG | Sucrose-phosphate synthase |
| 107762980 | CTTGGACACCAGCAGGAGGT | CAGCACAACCGCCAGACTTA | Beta-galactosidase |
| 107771686 | CATGTAGCCAGGAGGTCACG | CTATGTGTGGTCCGAGGAAGG | Beta-amylase, Chloroplastic-like |
| 107786350 | AGGATGAATCTGAGGTGGTGACA | CCAGAAGCCTCAATAGCCACA | Xylem cysteine proteinase |
| 107777405 | GCTCCTCTTATTCAATTCTCC | TTCGCTGCCATCATTCTT | Phosphoenolpyruvate carboxylase |
| 107766567 | AACAACAAGTCACCAGGATA | TATGCCTTCTTCGCCTCT | Ribulose bisphosphate carboxylase small chain, Chloroplastic |
| Gapdh | AGTGAACGACCCATTTATCT | AAAGACTCTGACGGACTTCT |  |

**References**

Livak K, Schmittgen T. Analysis of relative gene expression data using real-time quantitative PCR and the 2^-DDCT^ method. Methods.2001;25:402–408

Mortazavi A, Williams BA, McCue K, Schaeffer L, Wold B. Mapping and quantifying mammalian transcriptomes by RNASeq. Nat Methods. 2008;5:621–628

Reiner A, Yekutieli D, Benjamini Y. Identifying differentially expressed genes using false discovery rate controlling procedures. Bioinformatics. 2003;19:368–375

Shan Z, Wu HL, Li CL, Chen H, W Q. Improved SDS method for general plant genomic DNA extraction. Guangdong agricultural sciences. 2011;113-115
